# Effects of potassium on hydrothermal carbonization of sorghum bagasse

**DOI:** 10.1186/s40643-023-00645-4

**Published:** 2023-04-04

**Authors:** Shuhei Yoshimoto, Numan Luthfi, Kanta Nakano, Takashi Fukushima, Kenji Takisawa

**Affiliations:** Graduate School of Bioresources, Mie University, 1577 Kurimamachiyacho, Tsu, Mie 514-8507 Japan

**Keywords:** Hydrothermal carbonization, Potassium, Sorghum bagasse

## Abstract

Hydrothermal carbonization (HTC) reacts with biomass in water at a high temperature and pressure to produce hydrochar with a higher heating value (HHV) and lower ash content than dry torrefaction. The high potassium content in biomass can promote thermochemical conversion; however, it lowers the melting temperature of the ash, causing slugging and fouling. Therefore, this study, investigated the effect of potassium on the HTC of sorghum bagasse by comparing the removal of potassium by washing with the addition of K_2_CO_3_. Consequently, the ash content was the highest in the potassium-added hydrochar and was 3.81% at a reaction time of 2 h. Elemental analysis showed that the lower the potassium content, the higher the carbon content, and the hydrochar with potassium removed by water washing at a reaction time of 3 h had the highest carbon content at 68.3%. Fourier transform infrared spectrometer showed dehydration and decarboxylation reactions due to HTC, but no significant differences were observed between the potassium concentrations. The mass yield decreased with increasing potassium content, and was 27.2% for the potassium-added hydrochar after 3 h. This trend was more pronounced with increasing reaction temperature. On the other hand, HHV was not affected by the potassium content. Therefore, the energy yield was similar to the weight yield. Thermal gravimetry and derivative thermal gravimetry (TG-DTG) analysis showed that higher potassium tended to accelerate the decomposition of lignin and decrease the oxidation temperature.

**Graphical Abstract:**

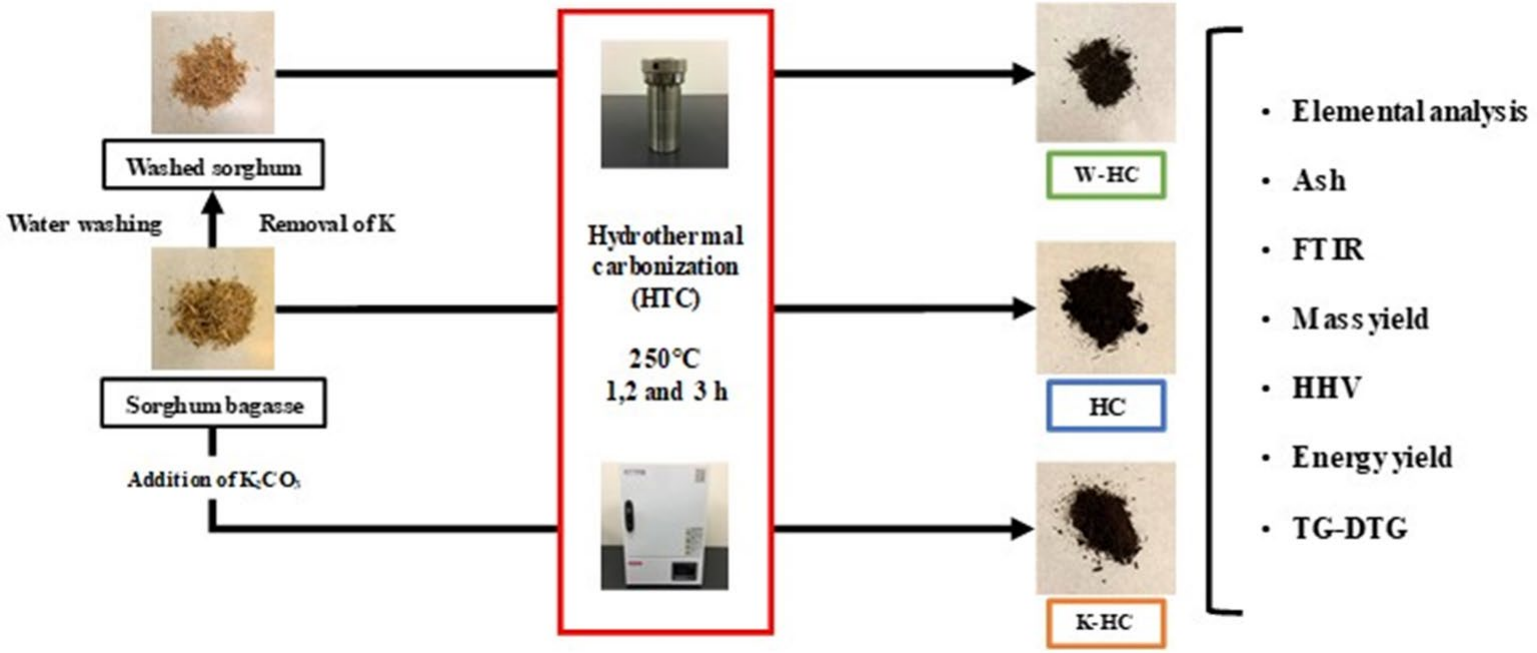

## Introduction

Climate change is one of the most important environmental topics in the world. The potential threat of global climate change associated with greenhouse gas emissions by the consumption of fossil fuels has significantly increased (Wuebbles and Jain 2001). In addition, fossil fuel is widely recognized as unsustainable as a primary energy source, and the depletion of fossil fuels may induce an energy crisis in the future (Khan et al. 2009). Thus, it is necessary to develop techniques to minimize the impacts of global warming caused by the increase in anthropogenic greenhouse gas emissions due to the excessive use of fossil fuels. Biomass utilization has been widely used to solve the environmental problem and biomass has attracted significant attention as a sustainable energy resource. Biomass can be combusted directly, to generate heat and power. However, raw biomass has some disadvantages, such as high moisture content, low calorific value, hygroscopic nature and low bulk density, which lead to low energy efficiencies and difficulties in its collection, grinding, storage, and transportation (Bridgwater 2006). Therefore, several biomass upgrading methods have been developed to overcome the above defects, such as the thermal, biological, mechanical, or physical processes.

Torrefaction is a biomass upgrading technology that has recently attracted considerable attention. Torrefaction is a thermal method that operates in the temperature range of 200–300 °C under atmospheric conditions in absence of oxygen (Stelt et al. 2011).It can generate biochar with a high energy density, low moisture content, high hydrophobicity, improved grindability and high uniformity (Bridgwater 2006). Biochar can serve as a fertilizer and soil conditioner (Keskinen et al. 2019), adsorbent, catalyst support (Shen 2015), carbon sequestration agent (Abdel-Fattah et al. 2015), and biofuel. In contrast, hydrothermal carbonization (HTC), also known as wet torrefaction, is a thermochemical conversion process with water at 180–265 °C under subcritical pressure (Acharya et al. 2015). Subcritical condition decreases the dielectric constant, weakens hydrogen bonds in water and produces high ionization constant, which improves the dissociation of water into acidic hydronium and basic hydroxide ions (Marcus 1999; Savage 1999). Subcritical water can also serve as a catalyst because of an increased hydrogen ion concentration (Ruiz et al. 2013). Furthermore, HTC can produce hydrochar and aqua phase (AP) and gases. The main product, hydrochar has higher heating value (HHV) compared to biochar produced by dry torrefaction, because subcritical water dissolves inorganic compounds in biomass. According to Reza et al. (2013), HTC treatment could remove up to 90% of calcium, magnesium, sulfur, phosphorus and potassium from biomass. HTC also saves pre-drying of the feedstock due to the improved dewaterability of hydrochar (Bach et al. 2017; Wang et al. 2018). The by-product AP contains abundant carbon and other elements, such as N, K, and P. Discharge of AP would result in wastage of these resources and, more importantly, lead to environmental contamination, e.g., water pollution and eutrophication. Therefore, many researches are being conducted on AP utilization methods, such as recycling by hydrothermal treatment, microalgae culture media and anaerobic digestion (Leng et al. 2020). The by-product gases produced in hydrothermal carbonization include carbon dioxide and carbon monoxide, as well as hydrogen and methane that can be used as energy (Hoekman et al. 2011). The gas produced by hydrothermal carbonization is soluble in AP and, therefore, produces less gas than traditional carbonization (González-Arias et al. 2022).

Therefore, this study investigates the effect of potassium existence on HTC. It is well-known that potassium is one of the most abundant metals in biomass. Previous researchers reported that potassium had a catalytic effect on the thermochemical conversion of biomass. Hwang et al. (2013) showed that potassium accelerated chemical reactions, such as dehydration and demethoxylation in fast pyrolysis of poplar wood. Safar et al. (2019) investigated the reactivity of biomass impregnated with potassium in torrefaction. They showed that at least 28% and up to 93% of time-saving was achieved using potassium-impregnated biomass in torrefaction. However, the presence of potassium reduces the ash melting point of the material, which may induce slugging and fouling in combustion (Du et al. 2014). Other researchers demonstrated that the combined water washing–torrefaction pretreatment could effectively remove inorganic matter and improve the fuel characteristics in the pyrolysis of rice husk via microwave heating (Zhang et al. 2015). Moreover, it is unclear how the presence or absence of potassium affects pyrolysis at an industry level. In addition, there are few reports that investigate the effect of the presence or absence of potassium on HTC.

Therefore, this study investigated the effect of the addition and removal of potassium on the HTC of sorghum bagasse to understand a suitable pretreatment for HTC. To achieve this, thermogravimetric analysis, elemental analysis, inorganic analysis, functional group analysis and calorimetric analysis were conducted using hydrochar derived from raw, potassium-added, and potassium-removed sorghum bagasse.

## Materials and methods

### Materials

In this study, sorghum bagasse supplied by the Farm Station of Mie University was selected as the feedstock. This raw material was first ground with a high-speed blender (YKB, AS ONE Co., Japan) and sieved to 4 mm. After that, the feedstock was completely dried at 105 °C for 24 h.

### Hydrothermal carbonization

To observe the effect of potassium on HTC, we conducted several experiments under three conditions: HTC, water washing HTC, and HTC with K_2_CO_3_ addition. HTC was conducted in a reactor (4744, Parr Instrument Company, USA) filled with 3.0 g of completely dried raw material and 30 mL of deionized water. The reactor was put in a constant temperature dryer (OF-300 V, AS ONE Co., Japan) at 250 °C and kept at the same temperature for reaction times. Reaction times were selected as 1, 2 and 3 h. After removing the reactor from the constant temperature dryer and cooling it to room temperature, the solid product was filtered with qualitative filter paper (5 µm, 285 dia., AS ONE Co., Japan) and completely dried at 105 °C for 24 h. A solid dried product was designated as HC.

Water washing HTC was conducted by washing with deionized water before the HTC. Water washing was performed by stirring 15 g of the raw material in 1500 mL of deionized water at 60 °C for 6 h to remove potassium. After that, washed sorghum bagasse was filtered with qualitative filter paper (5 µm, 285 dia. AS ONE Co., Japan) and completely dried at 105 °C for 24 h. Then, the sample was treated with HTC under the same conditions as the HTC and denoted as W-HC.

HTC with K_2_CO_3_ addition was conducted by modifying deionized water to 0.05 mol/L of an aqueous solution of K_2_CO_3_ under the same HTC conditions as the HTC. The sample was denoted as K-HC. Each experiment was conducted three times.

### Analysis

Inorganic analysis (Ca, K, Mg, Na, and P) of raw and washed sorghum bagasse were performed by inductively coupled plasma optical emission spectrometry (Agilent 5110 ICP-OES, Agilent Technologies, USA). Raw and washed sorghum were heated and dissolved in nitric acid and hydrogen peroxide as a pretreatment for inorganic analyses. The elemental analysis (C, H, N, and O) of the raw material and hydrochar was conducted using elemental analyzers (vario EL cube, Elementar, Germany). The ash content of each sample was determined based on the weight difference before and after the heating of the sample at 550 °C using a muffle furnace (HPM-OG, AS ONE Co., Japan) for 3 h and calculated as follows:

1 $$Ash\,(\mathrm{\%})= \frac{{W}_{a}}{{W}_{b}} \times 100$$

where *W*_a_ and *W*_b_ are the weights after and before combustion, respectively. Functional group analyses on the sample surface were confirmed from the records in the range of 4000–600 cm^−1^ using Fourier transform infrared spectrometer (FTIR) (Spectrum Spotlight200, PerkinElmer Co., USA). The mass yield was calculated as follows:

2 $$Mass\,yield \left(\mathrm{\%}\right)= \frac{{W}_{h}}{{W}_{f}} \times 100$$

where *W*_*h*_ is the hydrochar weight, *W*_*f*_ is the feedstock weight (Sermyagina et al. 2015). It is determined the weight of samples before and after HTC using an electronic balance. The higher heating value (HHV) of the samples was measured in accordance with standard procedure (JIS M8814) using a vacuum-type adiabatic calorimeter (O.S.K 150, Ogawa Sampling Co., Japan) involving a water equivalent of 322.22 J/°C. The water equivalent was the value obtained when the heat capacity of the calorimeter was converted to water; which was calculated using benzoic acid as a standard. The HHV was calculated as follows:

3 $$HHV{\text{~}}\left( {{{{\text{MJ}}} \mathord{\left/ {\vphantom {{{\text{MJ}}} {{\text{Kg}}}}} \right. \kern-\nulldelimiterspace} {{\text{Kg}}}}} \right) = \frac{{(332.22 + 1500) \times (T_{a} - T_{b} ) \times 4.2}}{{W_{{HHV \times }} 10^{3} }}$$

where *W*_*HHV*_, *T*_a_*,* and *T*_b_ are the sample weight, temperature after combustion, and temperature before combustion, respectively, after adding 1500 mL water into the calorimeter and measuring the temperature difference before and after the combustion of the sample. The energy yield was calculated as follows:

4 $$Energy\,yield \left(\%\right)=M\times \frac{{HHV}_{x}}{{HHV}_{0}}$$

where *M*, *HHV*_*x*_, and *HHV*_*0*_ are the mass yield, HHV of the sample, and HHV of the raw material, respectively. Thermal gravimetry and derivative thermal gravimetry (TG-DTG) analysis was performed using thermogravimeter-differential thermal analyzer (TG/DTA6200, Seiko Instruments Inc., Japan). In each case, 5 mg of sample was placed at the bottom of the alumina crucible and heated from 30 to 550 °C under a heating rate of 5 °C/min. The mass loss of the sample was measured at 6 s intervals.

## Results and discussion

### Elemental analysis and ash

**Fig. 1:**
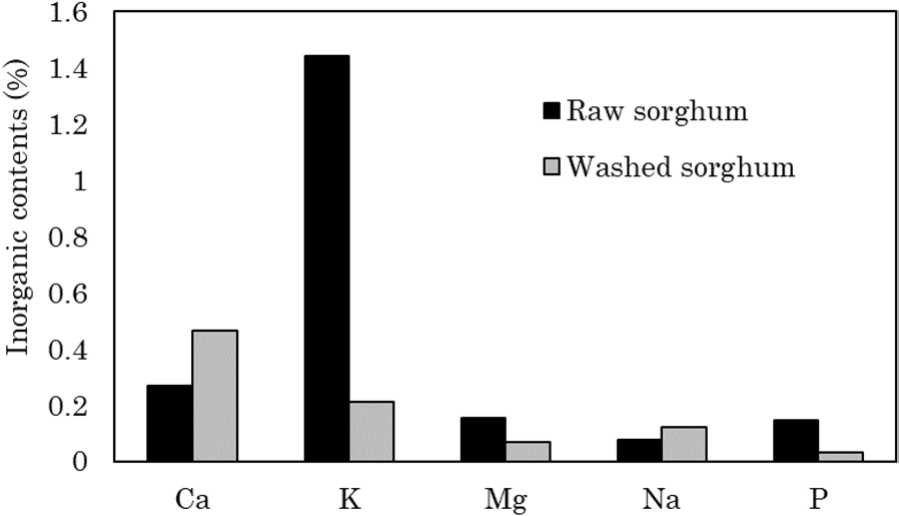
Percentage of inorganic substances in the raw and washed sorghum as determined by ICP analysis

Figure 1 shows the percentage of inorganic substances in the raw and washed sorghum bagasse as determined by ICP analysis. Sorghum bagasse contained the highest amount of potassium, which could be removed by about 80% by washing in water. Magnesium and phosphorus also decreased slightly, while calcium and sodium increased slightly in percentage. Previous studies have also reported that washing did not remove calcium, magnesium, or sodium, whereas potassium was removed significantly (Cen et al. 2016; Chen et al. 2017; Deng et al. 2013). In this experiment, the changes in inorganic substances other than potassium were small and did not seem to affect hydrothermal carbonization. Liaw et al. (2013) reported that organic matter is also leached out by washing in water, and the liquid after washing in water was tinted brown, indicating that organic matter, such as organic acids, may have been removed.

**Fig. 2:**
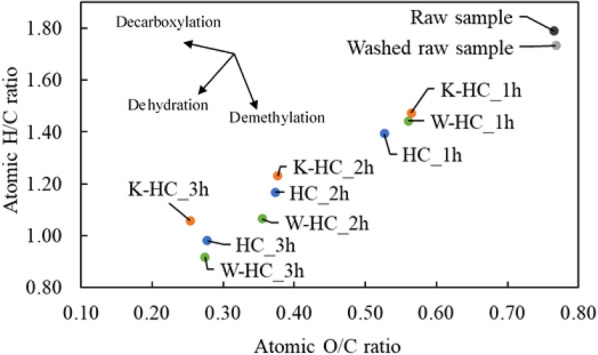
Atomic O/C and H/C ratios of raw sorghum, washed sorghum, and hydrochar

Table 1 shows the results of elemental analysis and ash content of the raw material and hydrochar. Compared with the raw sorghum, the percentage of carbon in the washed raw material increased and the ash content decreased significantly. Previous studies have reported significant removal of inorganic materials by water washing (Cen et al. 2016; Chen et al. 2017). The ash content was also significantly reduced in the HC and W-HC, which was positive for the fuel. This may be due to the dissolution of ash into the liquid phase during HTC. In HC, as the reaction increased, the ratio of carbon increased, and the ratios of hydrogen and oxygen decreased by removing hydroxyl groups through dehydration reaction and removing carboxyl and carbonyl groups through decarboxylation reaction (González-Arias et al. 2020). The same trend was observed at W-HC and K-HC. To further evaluate the changes in elemental composition, Fig. 2 shows graphs of atomic O/C and H/C ratios of the raw material, washed material, and hydrochar. The graph shows that the lower the left-hand side of the distribution, the better the hydrochar is as a fuel. Dehydration reactions decrease O/C and H/C ratios, decarboxylation reactions decrease O/C ratio and increase H/C ratio, and demethylation reactions increase O/C ratio and decrease H/C ratio (Reza et al. 2014). The O/C and H/C ratios of the hydrochar decreased as the reaction time increased due to decarboxylation and dehydration reactions. The H/C ratios of W-HC at 2 and 3 h were smaller than those of HC and K-HC, indicating that W-HC is a superior fuel. K-HC_3h had the lowest O/C ratio. Chen et al. (2021) reported that K_2_CO_3_ significantly accelerated the decarboxylation of cellulose at 150–250 °C in their pyrolysis experiments with K_2_CO_3_. Rutkowski (2011) reported a decrease in O/C ratio and enhancement of deoxygenation in pyrolysis with K_2_CO_3_. Similar results were observed in this experiment, suggesting that the decarboxylation reaction may have accelerated at 3 h, which is the most severe condition.

**Table 1:** Elemental analysis (dry basis) and ash of raw material and hydrochar

Sample	C [*w*/*w *%]	H [*w*/*w *%]	O [*w*/*w *%]	N [*w*/*w* %]	Ash [*w*/*w* %]	H/C	O/C
Raw sorghum	43.5 ± 0.3	6.48 ± 0.01	44.4 ± 0.3	0.45 ± 0.03	5.27 ± 0.4	1.79	0.77
Washed sorghum	45.1 ± 0.4	6.51 ± 0.03	46.2 ± 0.3	0.34 ± 0.02	1.92 ± 0.1	1.73	0.77
HC_1h	53.9 ± 0.2	6.26 ± 0.02	37.9 ± 0.3	0.60 ± 0.04	1.34 ± 0.2	1.39	0.53
HC_2h	60.9 ± 0.4	5.93 ± 0.00	30.3 ± 1.2	0.80 ± 0.05	2.03 ± 0.7	1.17	0.37
HC_3h	67.8 ± 0.5	5.54 ± 0.02	25.1 ± 0.6	0.60 ± 0.05	1.03 ± 0.1	0.98	0.28
W-HC_1h	52.7 ± 0.8	6.34 ± 0.01	39.4 ± 0.8	0.36 ± 0.07	1.16 ± 0.04	1.44	0.56
W-HC_2h	63.4 ± 0.5	5.63 ± 0.04	30.0 ± 0.3	0.40 ± 0.01	0.62 ± 0.1	1.07	0.36
W-HC_3h	68.3 ± 0.3	5.22 ± 0.02	25.0 ± 0.3	0.46 ± 0.02	1.01 ± 0.05	0.92	0.27
K-HC_1h	51.9 ± 0.4	6.37 ± 0.01	39.0 ± 0.4	0.31 ± 0.00	2.44 ± 0.07	1.47	0.56
K-HC_2h	59.6 ± 0.9	6.12 ± 0.04	30.0 ± 0.5	0.50 ± 0.03	3.81 ± 0.4	1.23	0.38
K-HC_3h	67.2 ± 1.0	5.92 ± 0.03	22.7 ± 0.8	0.68 ± 0.06	3.47 ± 0.3	1.06	0.25

### FTIR

**Fig. 3:**
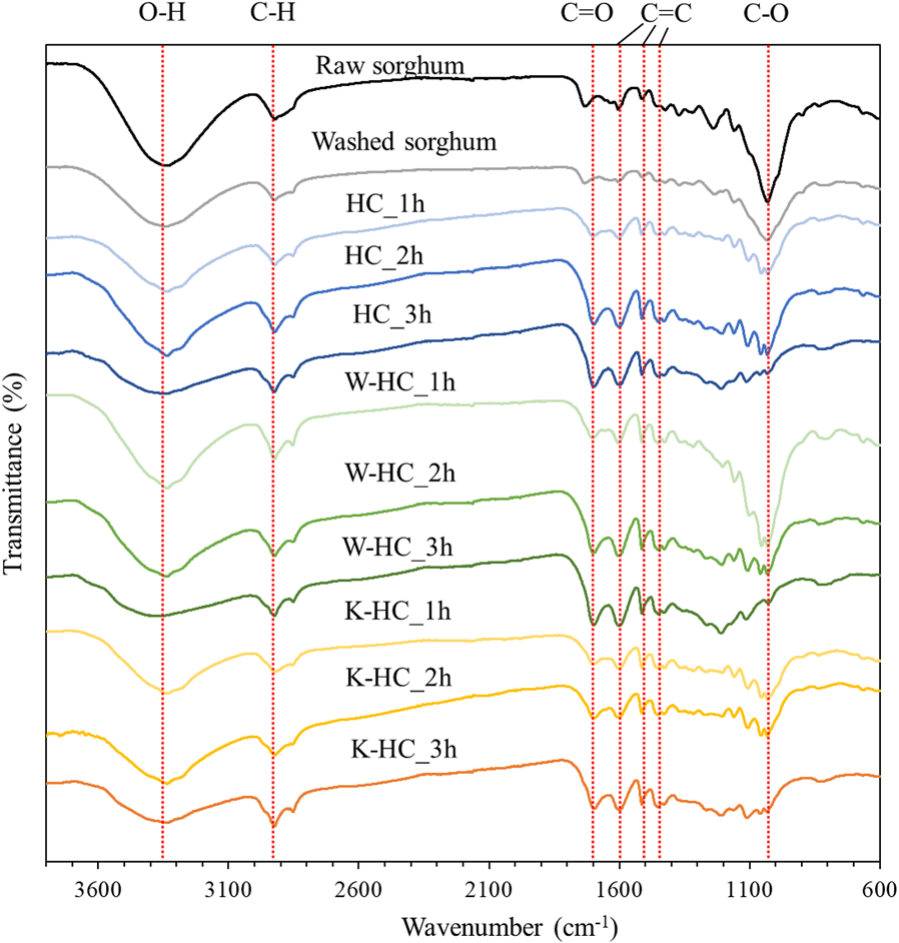
FTIR spectrum of raw material and hydrochar

Figure 3 shows the FTIR spectrum analysis; the 3350 cm^−1^ is due to O–H stretching vibrations of hydroxyl and carboxyl groups (Ma et al. 2015), with peaks in all samples. The peak was largest for raw sorghum, and slightly smaller for washed sorghum. Diao et al. (2021) also reported a similar reduction of the 3350 cm^−1^ peak by water washing. For the hydrochar, the peak decreased as the reaction time increased. This suggests that the dehydration reaction has progressed (Wang et al. 2017). The band at 2920 cm^−1^ corresponds to an aliphatic C–H stretching vibration, suggesting the presence of aliphatic and hydroaromatic structures. It was present in all samples and did not significantly change. The 1700 cm^−1^ peak is attributed to C=O bond stretching vibrations, with the peak increasing as the reaction time increases. The 1600 cm^−1^ peak is attributed to aromatic C=C bonds. Reza et al. (2014) reported that aromatization may occur during HTC. The peaks at 1508 and 1460 cm^−1^ are attributed to the C=C bond of lignin, and the peaks became sharper and larger with HTC, but did not change with the reaction time or potassium content. The peak at 1031 cm^−1^ is due to C–O bonding, which decreases as reaction time increases. The peak at W-HC_1h is almost the same as that of the raw material, indicating that the deoxygenation reaction had not progressed. Zhang et al. (2018) reported that this peak was larger than the raw material in the low temperature zone and became smaller as the temperature increased in their experiment on torrefaction after water washing, and the same trend was observed in this experiment.

### Mass yield

**Fig. 4:**
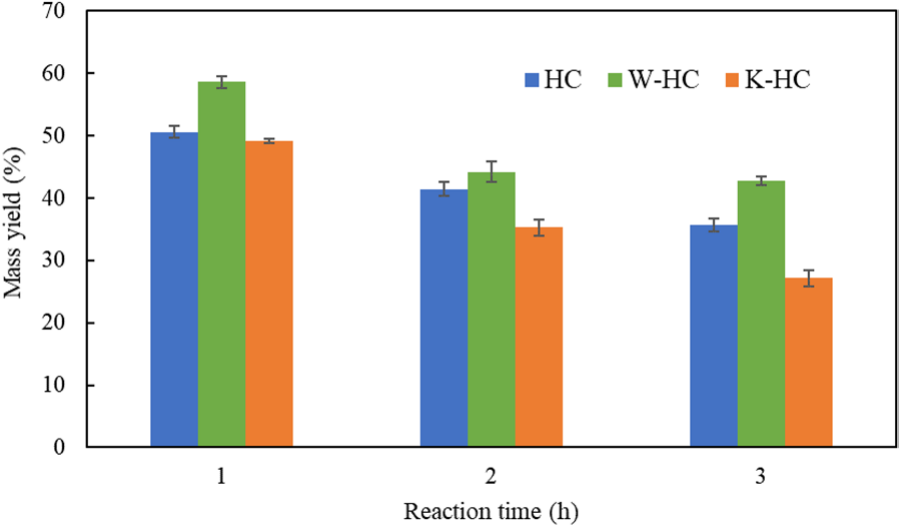
Mass yield of hydrochar at 250 °C

Figure 4 shows the mass yield results of hydrochar. The mass yield decreased as the reaction time increased, and was higher at W-HC than at the other conditions. In contrast, K-HC gave the smallest mass yield. Previous studies have reported that the mass yield in pyrolysis decreases with increasing potassium content (Safar et al. 2019; Yafei et al. 2020). In this experiment, the mass yield in W-HC increased, because more potassium was removed by washing in water. Moreover, the mass yield in K-HC decreased because of the addition of K_2_CO_3_. It is known that the cations of alkali salts catalyze aqueous gas formation reactions; thus, promoting gas formation instead of individual products. Correa et al. (2019) reported that the final pressure in the reactor was greater for samples containing more alkali metals in their HTC experiments.

### HHV and energy yield

**Fig. 5:**
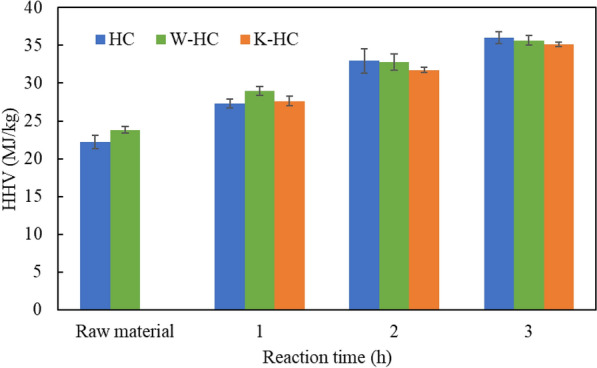
HHV of raw material, washed sorghum, and hydrochar at 250 °C

Figure 5 shows the HHV of raw sorghum, washed sorghum, and hydrochar. The HHV in washed sorghum increased by water washing. Chen et al. (2017) reported similar results due to a decrease in ash content, an increase in the proportion of carbon, and an increase in combustible components. The HHV increased as the reaction time of HTC increased. However, no significant difference was observed in HHV among HC, W-HC, and K-HC. Zhang et al. (2022) reported that torrefied K_2_CO_3_ and microalgae in a 1:1 ratio increased HHV from 25.0 to 27.1 MJ kg^−1^ at 200 °C, 25.8 to 28.7 MJ kg^−1^ at 250 °C, and 26.4 to 31.9 MJ kg^−1^ at 300 °C. It is possible that in this experiment, the HHV also increases as the concentration of K_2_CO_3_ increases.

**Fig. 6:**
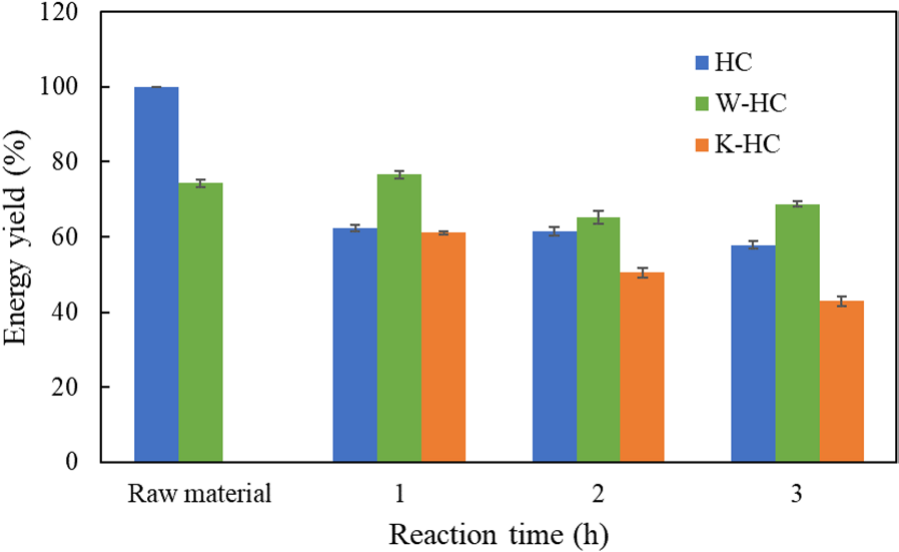
Energy yield of raw material, washed sorghum, and hydrochar at 250 °C

Figure 6 shows the energy yield of raw material and hydrochar. The energy yield of W-HC was the largest at all reaction times, indicating that W-HC is the most suitable for fuel among other reactions. In contrast, the energy yield of K-HC was the smallest among other reactions, indicating that the lower mass yield affected the energy yield.

### TG-DTG

**Fig. 7:**
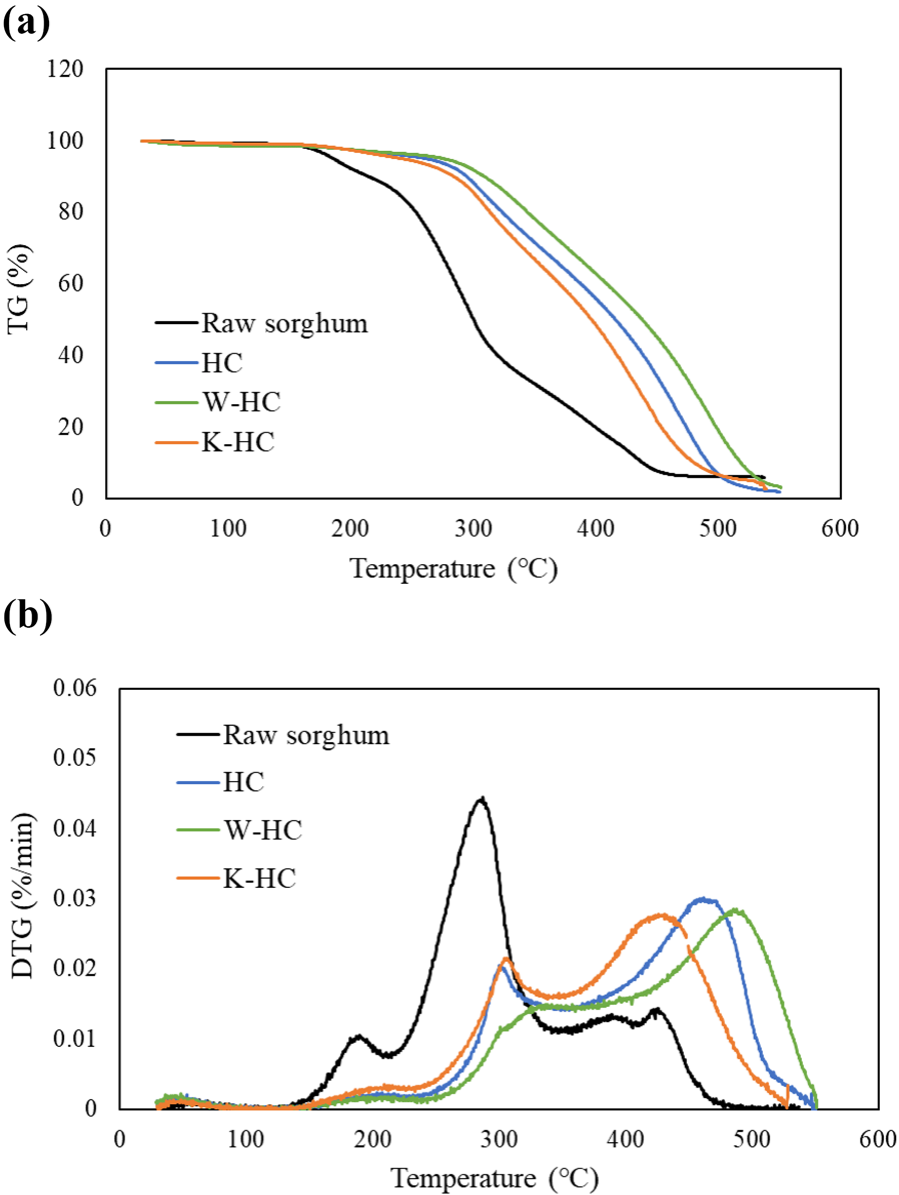
**a** TG of raw sorghum and hydrochar at 250 °C for 3 h. **b** DTG of raw sorghum and hydrochar at 250 °C for 3 h

Figure 7 shows the TG and DTG curves of raw sorghum and hydrochar reacted at 250 °C for 3 h. The main oxidation and combustion reactions occurred between 200 °C and 500 °C. The first peak near 200 °C in the DTG curve is due to hemicellulose, and the second peak near 300 °C is due to the decomposition of cellulose and the partial decomposition of lignin. The peak around 400–500 °C is mainly due to char oxidation, resulting from the combustion of lignin or fixed carbon in the sample (Ferreira et al. 2021). HTC eliminated the first peak around 200 °C, indicating that the hemicellulose was greatly degraded by HTC. This phenomenon was also reported by PetroviĿ et al. (2016) in the hydrothermal treatment of grape pomace. The second peak, around 300 °C, was reduced by HTC, indicating that cellulose was decomposed by HTC. It was particularly reduced at W-HC and shifted toward higher temperatures. This may be due to the removal of alkaline earth metals, such as potassium, by water washing, which did not promote combustion (Deng et al. 2013). The third peak, around 400–500 °C was larger due to HTC. This may be due to the repolymerization of lignin after its decomposition. The third peak shifted to lower temperatures as the potassium concentration increased. It is well-known that K_2_CO_3_ accelerates the lignin decomposition (Xu et al. 2019), lowers the oxidation temperature, and increases the combustion reaction rate (An et al. 2006; Jones et al. 2007).

## Conclusions

This study investigated the effect of potassium on HTC by varying the content of potassium by washing and addition. W-HC with potassium removed by water rinsing had a higher percentage of carbon, a higher mass yield, and a higher energy yield. It was found that the K-HC with K_2_CO_3_ addition enhanced the decomposition reaction of HTC, and the mass yield was smaller than that of the other conditions. However, HHV and FTIR spectra did not change significantly with potassium concentration. TG-DTG analysis suggested that the higher potassium content promoted the lignin decomposition and lowered the oxidation reaction temperature.

## Data Availability

All data analyzed during this study are included in this article.

## Abbreviations

HTC: Hydrothermal carbonization
HC: Hydrochar
W-HC: Hydrochar produced by hydrothermal carbonization after water washing
K-HC: Hydrochar produced by hydrothermal carbonization with K_2_CO_3_ addition
HHV: Higher heating value
FTIR: Fourier transform infrared spectrometer
TG: Thermogravimetry
DTG: Derivative thermogravimetry
